# From Waste to Wealth: Covalent Organic Frameworks for Gold Detection and Recovery from Secondary Sources

**DOI:** 10.1021/acsami.5c09740

**Published:** 2025-08-28

**Authors:** Salma Abubakar, Asmaa Jrad, Gobinda Das, Thirumurugan Prakasam, Samer Aouad, Mark A. Olson, Ali Trabolsi

**Affiliations:** † Science Division, Chemistry Program, 167632 New York University Abu Dhabi, Saadiyat Island, 129188 Abu Dhabi, United Arab Emirates; ‡ Water Research Centre, 167632 New York University Abu Dhabi, Saadiyat Island, 129118 Abu Dhabi, United Arab Emirates; § Department of Chemistry, Faculty of Arts and Sciences, University of Balamand, P.O. Box 100 Tripoli, Lebanon; ∥ Department of Physical & Environmental Sciences, 14738 Texas A&M University Corpus Christi, 6300 Ocean Dr., Corpus Christi, Texas 78412, United States

**Keywords:** adsorption, covalent organic frameworks, detection, gold recycling, gold waste, sustainability

## Abstract

Gold is a precious element, renowned for its diverse applications in catalysis, biomedicine, and electronics, largely due to its remarkable stability and superior conductivity. However, the escalating global demand and intensive mining activities have prompted a shift toward exploring gold recovery from alternative and secondary sources. Traditional gold recovery techniques, such as hydrometallurgy and cyanidation, are efficient yet notorious for their toxic byproducts, necessitating the pursuit of more sustainable methods. This review explores the potential of covalent organic frameworks (COFs) as cutting-edge materials for gold detection and adsorption. COFs are distinguished by their precise architecture, inherent porosity, and customizable functionalities, rendering them exceptionally suited for the selective capture of gold. First, we present an overview of the fundamental COF gold adsorption mechanisms, including coordination chemistry, hydrogen bonding, electrostatic interactions, and reduction processes. This is followed by examining COF synthesis methods, functionalization techniques, and composite engineering strategies that optimize their stability and adsorption efficiency. The review further highlights recent advancements in the utilization of COFs for gold sensing, recovery from electronic waste, and adsorption at trace concentrations. Finally, we address the current challenges in the application of COFs in this domain and propose future research directions. This comprehensive review serves as an invaluable resource for advancing gold extraction through COF-based materials, ultimately contributing to innovative and sustainable gold recovery practices.

## Graphical abstract

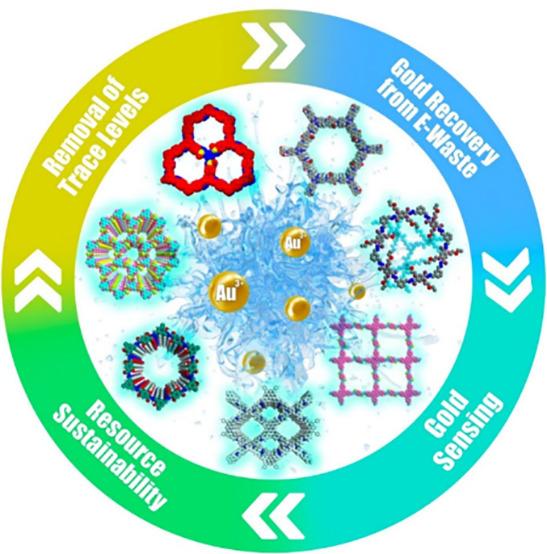

## 1 Introduction

Gold has been valued throughout human history, symbolizing wealth and playing a pivotal role in trade and the stability of the global financial market. Beyond its cultural and economic significance, gold is integral to various consumer-related and industrial applications, including jewelry and ornament production,^1,2^ electronics,^3–5^ alloy production,^6–8^ catalysis,^9,10^ and dental implants.^11–13^ The versatility of gold stems from its exceptional properties, such as malleability,^14^ high electrical conductivity,^15,16^ corrosion resistance,^17,18^ and biocompatibility.^19,20^ This leads to high global gold demand and consumption, resulting in extensive mining. Over the past decade, more than 38,000 tonnes of gold, worth over 3 trillion US dollars, have been extracted.^21^ In 2023 alone, gold consumption exceeded 4000 tonnes.^21,22^

Large-scale gold mining has severe environmental consequences, including land degradation and deforestation from heavy machinery.^23^ Furthermore, excessive gold mining leads to soil and water pollution from released heavy metals and other contaminants.^24^ These pollutants present serious health risks, such as neurological and kidney damage, along with other severe diseases.^25,26^ Moreover, the rapid depletion of natural gold reserves is a growing concern, as it is estimated that the mineral resource could be exhausted in approximately 150 years based on current extraction and consumption rates, according to the US Geological Survey.^27^ Additionally, mining and processing of gold result in it being released into water bodies, including wastewater and seawater, at levels below 10 μg L^–1^ and 10 ng L^–1^, which makes them valuable gold sources.^28^ However, at such diluted concentrations, the spent metal is largely inaccessible and thus challenging to recover. This emphasizes the urgent need to establish gold extraction and recycling strategies, as they play a crucial role in promoting gold sustainability and conservation.

A significant portion of mined gold is used in electronic equipment, where gold content usually ranges from 10 to 10,000 g per ton, while mobile devices can contain over 200 g per ton^29,30^ As a result, electronic waste (e-waste) has emerged as the prime secondary source of gold.^31^ An estimated 40–50 million tons of e-waste is generated annually worldwide, and this figure is expected to increase by 5 million tons each year.^32^ Generally, various technologies have been employed for gold extraction, including pyrometallurgy,^33,34^ hydrometallurgy,^35,36^ bio-oxidation,^37,38^ ion exchange,^39,40^ and cementation.^41,42^ However, these methods suffer from significant drawbacks, including limited gold selectivity, high operational costs, substantial energy demands, and the generation of secondary pollutants, such as cyanide and mercury residues.^31,43^ In contrast, adsorption techniques offer a simpler and more environmentally friendly alternative.^44^ Commonly used adsorbents such as activated carbon,^45,46^ organic polymers,^47,48^ and biomass materials^49,50^ have been extensively studied for gold ion adsorption. However, these conventional adsorbents usually exhibit slow uptake kinetics, poor selectivity, and low adsorption capacity.^51–53^ These limitations underline the necessity for advanced adsorbents that offer higher efficiency and selectivity. Recently, covalent organic frameworks (COFs) have garnered significant attention due to their exceptional versatility and tunable properties, making them highly promising adsorbents.

COFs are crystalline porous organic materials formed from covalently linked organic molecules, and they are known for their well-defined topologies, permanent porosities, ordered networks, and structural tunability.^54,55^ These properties enable the design of COFs for different applications, including sensing,^56^ energy storage,^57^ gas separation,^58^ CO_2_ conversion,^59^ drug delivery,^60^ catalysis,^61^ and wastewater treatment.^62,63^ Regarding adsorption, COFs possess key advantages of tailorable functionality and superior chemical stability over other porous materials, such as metal–organic frameworks (MOFs), which improve their selectivity and durability as adsorbents.^64^ In recent years, several COFs have displayed the ability to selectively detect and capture gold ions from water, making them promising candidates for effective gold recovery.^65–67^

This review provides a comprehensive overview of COFs and COF-based composites reported for gold adsorption, detection, and recovery from e-waste leachates and other aqueous media. First, we discuss the fundamental interaction mechanisms between COFs and gold ions, including coordination bonding, hydrogen bonding, and electrostatic interactions. These mechanisms play a crucial role in determining the adsorption selectivity and efficiency. Then, the design and polymerization strategies used to synthesize COFs tailored for gold recovery, such as Schiff base chemistry, keto-enamine, hydrazone, and carbon linkages, are highlighted. This is followed by a discussion on the contribution of COF functionalization to adsorption performance and metal capture from ultralow gold concentrations. Subsequently, we discuss key challenges facing COF-based gold recovery technologies, which include scalability and applicability on an industrial level, before outlining the future directions for advancing COF-based gold recovery technologies. Overall, this review sheds light on the potential of COFs to transform gold resource management by offering a highly selective, efficient, and eco-friendly alternative to traditional gold extraction methods.

## 2 Factors Influencing Selective Gold Capture

The process of gold ion adsorption via COFs for the recovery of the precious metal can be influenced by the COF’s surface area and pore architecture, which impact the diffusion of gold ions and their accessibility to the adsorption sites.^68,69^ Another factor is the COF surface and pore functionalization, which can determine the physical and chemical interactions with the analyte ions.^70^ As discussed in the next section, these interactions largely govern the fundamental mechanisms involved in the adsorption process and contribute to COF selectivity toward gold. Solution conditions, such as pH and the presence of competing ions, also affect gold adsorption efficiency. The solution pH influences the COF functional groups and the speciation of gold ions, as Au^3+^ ions are generally stable under acidic conditions.^71^ Understanding and analyzing these factors is critical for optimizing gold adsorption and contributes to overall metal resource conservation efforts.

## 3 Gold Adsorption Mechanisms

With knowledge of the interactions responsible for gold ion-COF binding, researchers can strategically design the materials to achieve enhanced uptake rates, capacity, and selectivity. The widely reported mechanisms responsible for gold uptake include coordination bonding, electrostatic interactions, and hydrogen bonding, each contributing uniquely to the overall adsorption.

### 3.1 Coordination Bonding

Coordination bonding, which involves electron sharing between metal ions and electron-rich ligands or atoms such as sulfur, nitrogen, and oxygen, is one of the strongest interactions that contribute to gold adsorption in COFs.^72,73^ This bonding is the foundation of many chemisorption processes that enable gold uptake from water based on the principle of hard and soft acids and bases (HSAB theory). The gold ions act as soft acids and electron acceptors that exhibit a strong affinity for the electron-donor ligands, which function as soft bases.^74^ The literature reveals that sulfur-based groups, such as thiols, thiones, and thioethers, coordinate strongly with gold. For instance, the binding constants of gold nanoparticles (AuNP) with thione and thiolate-based ligands can exceed 2 × 10^6^ M^–1^.^75^ Thiourea-based molecules have a binding constant for Au^3+^ greater than 7 × 10^6^ M^–1^.^76^ Thus, sulfur-based units are often introduced into COFs designed for gold recovery. Recent studies have reported sulfur-containing COF materials, namely MoS_2_-TpTa COF composites, in which Au^3+^ strongly coordinates with the thiol groups.^77,78^ Similarly, TTASDFP COF and TTB-COF, which incorporate anchored thioanisole and thioether groups, have demonstrated the ability to effectively capture Au^3+^ from trace levels in water.^67,79^ Similarly, COF adsorbents functionalized with nitrogen and oxygen groups, such as amines and hydroxyls, can also function as effective Au^3+^ adsorbents due to coordination bonding. This was reported in the examples of JNU-1 and TY-Hz COFs, which contain amide groups,^65,80^ MTpPa-1, which contains oxygen groups from the ketone units,^81^ and TpTsc COF rich with nitrogenous and hydroxyl groups.^82^ Moreover, to optimize the gold uptake efficiency, a combination of the previously discussed atoms can be incorporated, similar to MoS_2_-TpTa COF composites.^77^ In this study, the researchers incorporated N, O, and S atoms through imine, hydroxyl, and thiol groups, respectively, all of which coordinate with Au^3+^. These reports showcase the role of gold ion coordination in improving the gold adsorption performance of COFs.

### 3.2 Electrostatic Interactions

Aside from coordination bonding, electrostatic interactions are another major contributor to gold ion adsorption. These interactions arise from the attractive forces between gold ions and charged or polar units within COFs, such as carbonyls and hydroxyls.^83^ These interactions are affected by the COF’s surface charge and functionalization, the gold speciation, and the overall charge of the metal complex.^84^ Although there are various gold oxidation states, ranging from – 1 and +5, Au^+^ and Au^3+^ coordination compounds are generally considered the most stable and thus the most explored in research studies, with Au^3+^ being frequently investigated in adsorption studies.^85,86^ The gold ion speciation is influenced by several factors, including pH and the presence of other molecular or ionic ligands in the water system. For instance, Au^+^ can form complexes with thio-based ligands, such as Au (HS)_2_^–^, in near-neutral hydrothermal solutions (Figure 1),^87–89^ On the other hand, Au^+^ mainly forms Au­(OH)_(aq)_ complexes in alkaline solutions below pH 12, and can form [Au (NH_3_)_2_]^+^ in the pH range of approximately 4–10.^90^ Comparingly, Au^3+^ forms complexes with chloride ions in acidic and brine systems, with the square-planar [AuCl_4_]^−^ being a common example present in pH levels below 4 (Figure 1).^71^ In alkaline conditions, however, Au^3+^ can form [AuCl­(OH)_3_]^−^ and [Au­(OH)_4_]^−^ complexes under pH ranges of 5–6 and ≥7, respectively.^85^ In addition to pH, the surrounding solvent also affects the gold speciation. For instance, Au^+^ and Au^3+^ can form coordinate bonds with halides in alcohols, pyridine, DMSO, and other nonaqueous solvents.^86,91^

**Figure 1:**
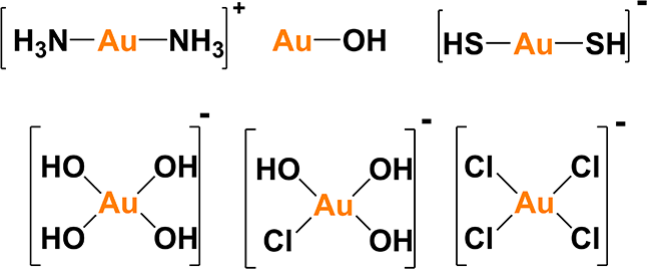
Structure of common gold-based complexes under different pH values and ligands.^90^

The negatively charged gold complexes bind to positively charged units of the COF via electrostatic interactions. The strength of these interactions is also influenced by the solution pH and the adsorbent’s surface charge. For instance, the cationic TpTG_Cl_ COF exhibited an approximately 50% decrease in the Au^3+^ adsorption capacity at pH levels of 7–9 compared to more acidic conditions (pH 2–3) due to a decrease in the zeta potential of the COF and the subsequent weakened electrostatic interactions with the gold ions.^92^ Similarly, COFs rich in ionic functional groups such as ionic-COF-Cl, demonstrated enhanced gold adsorption via electrostatic interactions.^66^ The cationic protonated nitrogen sites in the framework backbone served as primary adsorption sites for [AuCl_4_]^−^ anions, facilitating their capture from the solution.^66^ These findings highlight the impact of COF surface charge on the overall electrostatic interactions that lead to augmented gold adsorption efficiencies.

### 3.3 Hydrogen Bonding

Hydrogen bonding (H-bonding) is another key interaction that aids the adsorption of gold ions from water. This noncovalent bond occurs between a hydrogen covalently bonded to an electronegative donor atom (X) and another electronegative acceptor atom or group (X—H···Y).^93^ In the case of [AuCl_4_]^−^, the chloride components act as H-bond acceptors and can form bonds with hydroxyls (OH), and amine (N—H) functional groups in the COF materials, which supports the adsorption of the metal ions.^94^ The strength and selectivity of the H-bonding involving [AuCl_4_]^−^ is generally affected by the electronegativity difference of the hydrogen-donor atom, the donor–acceptor atom bond distance, the bond angle, the solution environment, and the potential formation of multiple H-bonds between the COF and [AuCl_4_]^−^.^95,96^ The impact of these factors on the selective [AuCl_4_]^−^ adsorption was best illustrated with JUU-1 COF, in which different H-bonds occur within the COF system involving four chloride atoms of [AuCl_4_]^−^ at the C=OH^+^, C=NH_2_^+^, and N—H sites. These H-bonding distances ranged between 2.25 and 3.05 Å, which fall within the range of expected strong H-bonding (Figure 2).^80,95^

**Figure 2:**
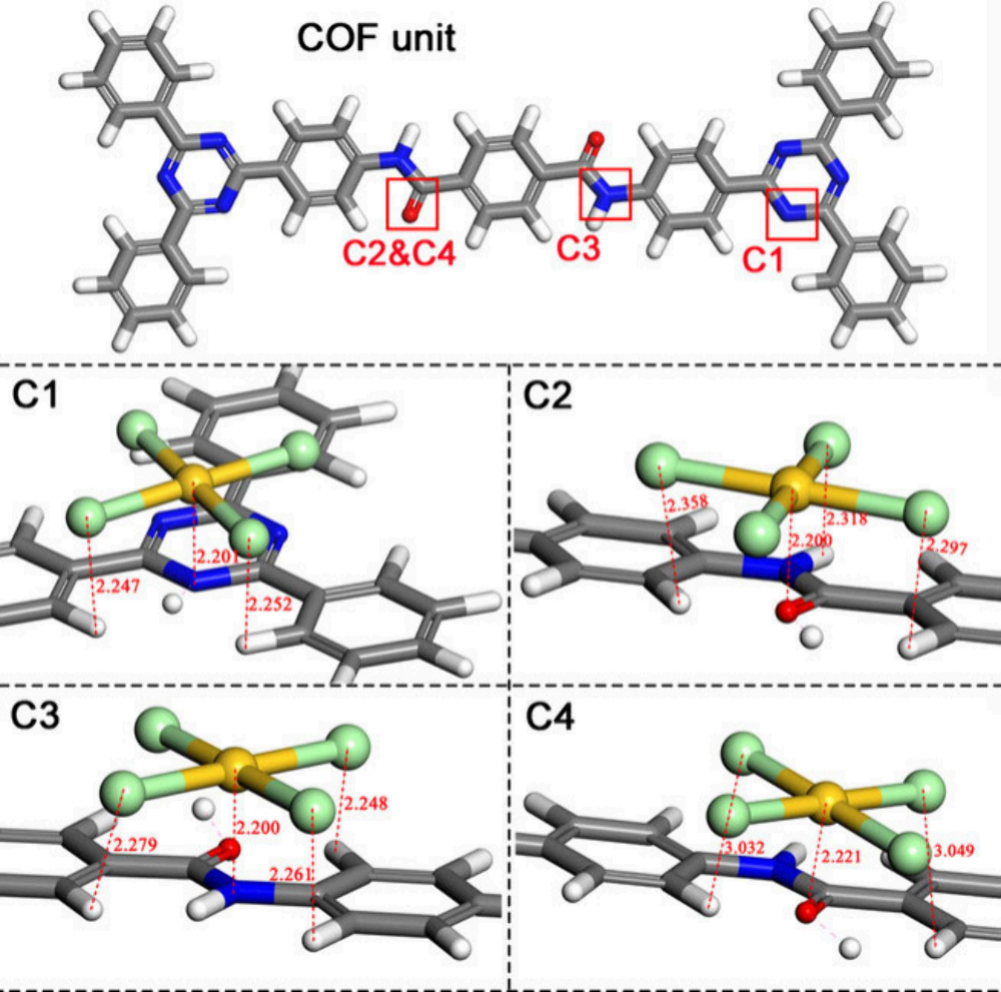
Structural configurations showcasing the sites (C1–C4) at which hydrogen and coordinate interactions take place between [AuCl_4_]^−^, JNU-1 COF. Adapted with permission from ref 80. Copyright 2020 Wiley.

The important role of H-bonding in gold recovery was also demonstrated with COF-HNU25, where the presence of phenolic OH groups in the pores, which acted as H-bond-based nanotraps, led to a higher gold uptake compared to the OH-free and isostructural COF-42.^94^ Moreover, recent studies showed that H-bonding can act in synergy with other noncovalent interactions, such as coordination, contributing to the overall enhanced selectivity toward [AuCl_4_]^−^. For instance, one study reported that hydrogen bonding involving protonated amides in TY-Hz COF (C=OH^+^···Cl and N—H···Cl), combined with O and N groups coordinated with [AuCl_4_]^−^, were integral to the mechanism behind the material’s gold sensing and uptake capabilities (Figure 3).^65^ These findings highlight the significant role of H-bonding in gold adsorption through COFs.

**Figure 3:**
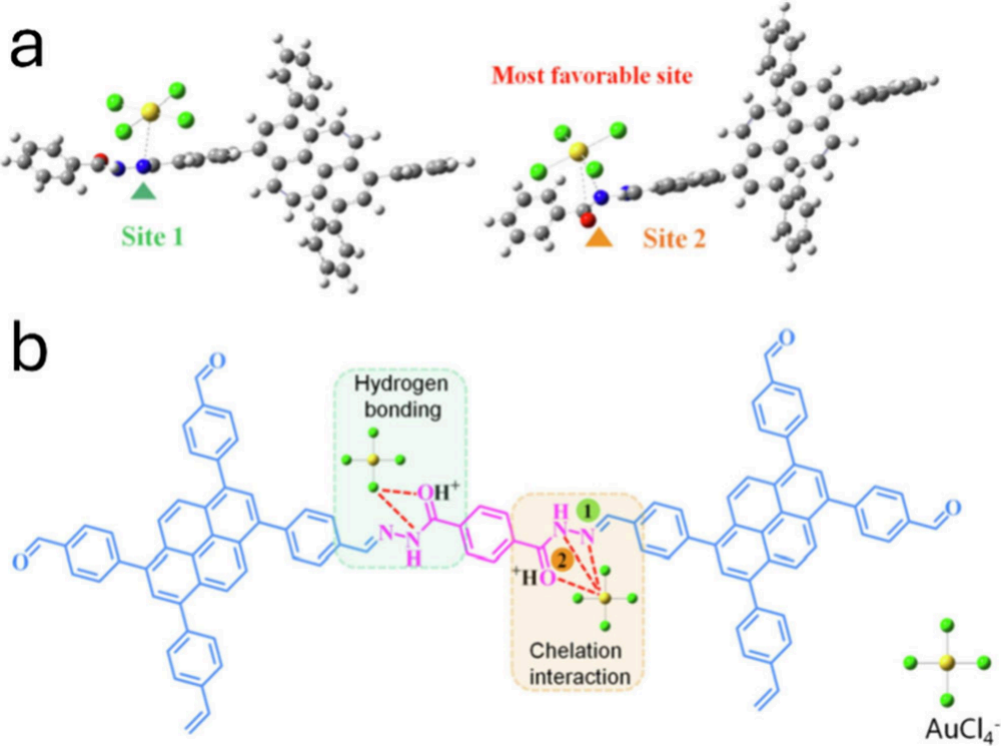
a) The structure of TY-Hz COF showing the gold ion binding sites. b) Hydrogen bonding and electrostatic interactions between the COF and [AuCl_4_]^−^. Adapted with permission from ref 65. Copyright 2023 Elsevier.

### 3.4 Gold Reduction

In addition to the previous interactions, some COFs can facilitate the reduction of gold ions to metallic Au^0^, which impacts the adsorption behavior, as metallic gold adheres to the adsorbent’s surface.^77,97^ This mainly occurs because the COF reduction potential is lower than that of gold ions and thus, they act as reducing agents. For instance, the reduction potential of TpTG_Cl_ COF was recorded at – 1.16 V vs a normal hydrogen electrode (NHE). On the other hand, the redox potentials for [AuCl_4_]^−^→[AuCl_2_]^−^ and [AuCl_4_]^−^→Au^0^ were measured at 0.926 and 1.002 V vs NHE, respectively.^92^ Gold reduction during adsorption was also reported with MoS_2_-TpTa COF, where the reduction of the ions is driven by sulfur oxidation, reducing Au^3+^ to Au^+^ and finally to metallic Au.^77^ Similarly, COF adsorbents functionalized with nitrogen and oxygen groups, such as amines and hydroxyls, also enable the reduction of Au^3+^ to Au^0^ during adsorption, as reported in the studies on JNU-1 COF, ionic-COF-Cl, TY-Hz COF, TpTsc COF, MTpPa-1, and TzDa-COF.^65,66,81,82^ Another mechanism for Au^3+^ reduction is photoreduction, which was reported for the isomers of Tp-BTD COF.^71^ In this example, the COF generates electron–hole pairs under visible light excitation, facilitating electron transfer and the reduction of the adsorbed [AuCl_4_]^−^.^98^ All of these reports showcase the synergistic impact of gold ion reduction within COFs on their gold removal performance. A major advantage of gold reduction is the possibility of recovering the metallic gold with high purity, making it a valuable recycled material. This was reflected in the TTB-COF study, where gold was obtained via pyrolysis, a thermal decomposition process in which the COF is subjected to high temperatures (Figure 4).^67^

**Figure 4:**
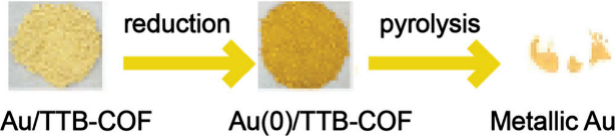
Recovery of metallic gold after adsorption via TTB-COF followed by pyrolysis. Reproduced with permission from ref 67. Copyright 2018 Royal Society of Chemistry.

### 3.5 Assessing the Mechanistic Contributions

While understanding the principles of the different mechanisms involved in gold adsorption is essential, it is equally important to evaluate their relative mechanistic contributions. By identifying the dominant mechanisms under specific conditions and the factors influencing their mechanistic hierarchy, the design and performance of the COF materials can be optimized.^99^ Gold adsorption mechanisms can be ranked based on the strength of the COF-gold interactions. Coordinative interactions are typically among the strongest, with binding energies ranging from 50 kJ/mol to 300 kJ/mol, generally exceeding those of ion-dipole interactions (50- 200 kJ/mol).^100^ In contrast, hydrogen bonding exhibits a broader range of strengths (15–120 kJ/mol), with values above 60 kJ/mol considered strong.^95,100^ For COF-Au^3+^ systems, interaction strength depends on the nature and positioning of COF functional groups, the distances between Au^3+^ and COF binding sites, and adsorption conditions such as pH and ionic strength.^101,102^ The dominance of a given noncovalent interaction is also governed by the solution environment. For instance, low pH conditions promote the protonation of the adsorbates and COF functional groups, enhancing H-bonding and electrostatic interactions during the adsorption process.

## 4 COF Synthetic Methods: Tailoring Structures for Gold Recovery

Strategic COF design is crucial for optimal gold adsorption. One of the pivotal aspects of the design is the covalent linkages since they affect the structural stability, porosity, and functionalization characteristics of the frameworks, all of which contribute to gold adsorption. Thus, judicious selection of the type of polymerization chemistry applied is a key factor in material design. Based on the type of covalent bonds formed between the organic linkers (Figure 5a), COFs reported for gold adsorption can be categorized into imine,^79^ ketoenamine,^71^ hydrazone,^65^ and carbon-linked frameworks.^103^

**Figure 5:**
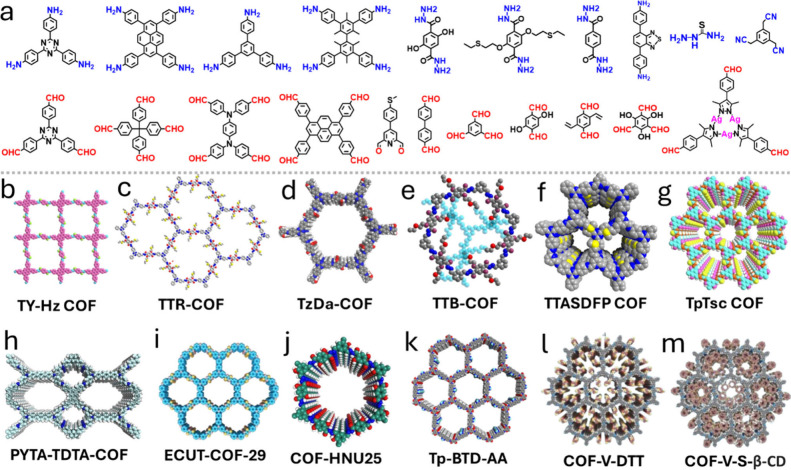
a) Structures of building blocks used to synthesize COFs. b–m) Examples of COFs reported for the adsorption and recovery of gold ions from water. b) Adapted with permission from ref 65. Copyrights 2023 Elsevier. c) Adapted with permission from ref 113. Copyright 2019 American Chemical Society. d) Adapted with permission from ref 109. Copyright 2023 Elsevier. e) Adapted with permission from ref 67. Copyright 2018 Royal Society of Chemistry. f) Adapted from ref 79. Available under a CC-BY 4.0 license. Copyright 2025 Abubakar et al. g) Adapted with permission from ref 82. Copyright 2021 Elsevier. h) Adapted with permission from ref 110. Copyright 2024 John Wiley and Sons. i) Adapted with permission from ref 22. Copyright 2024 Royal Society of Chemistry. j) Adapted with permission from ref 94. Copyright 2023 John Wiley and Sons. k) Adapted with permission from ref 71. Copyright 2022 American Chemical Society. l,m) Adapted with permission from ref 78. Copyright 2024 Elsevier.

### 4.1 Schiff Base Chemistry

Schiff base chemistry, which involves the reaction between primary amines and carbonyl compounds, is one of the most widely utilized approaches for COF synthesis.^104,105^ This reaction is favored due to the wide range of molecular precursors and tunable conditions used for the synthesis.^106^ As a result, COFs synthesized via Schiff base chemistry have become promising gold adsorbents.

#### 4.1.1 Imine Linkages

Imine-linked COFs, characterized by forming —C=N bonds, represent the largest group of COFs synthesized through Schiff base chemistry.^106^ In addition, they exhibit moderate chemical stability when exposed to water and various other organic solvents, and can be synthesized using different techniques, including solvothermal and microwave irradiation methods.^107,108^ As a result, imine-based COFs, such as TpTSc and TzDa-COF, have been explored for gold recovery (Figure 5d,g). TpTSc, developed by Zhang et al.,^82^ was formed through the solvothermal condensation of 2,4,6-triformylphloroglucinol (Tp) and alkyl amine thiosemicarbazide (Tsc). The COF was designed with thione, amino, and hydroxyl groups, which served as effective AuCl_4_^–^ binding sites. TzDa-COF,^109^ on the other hand, is a hydroxyl-rich COF synthesized by the Shi group via the solvothermal reaction of a triazine-based amine and a dihydroxyl-functionalized aldehyde. COF-HNU25 (Figure 5j), which was developed by Dr. Wang’s group, is another imine-based COF designed for gold recovery.^94^ The material was formed through the condensation of trimesaldehyde and derivatives of terephthalohydrazide. This study showcased the correlation between the ratio of phenolic hydroxyls in the material and the gold uptake efficiency. The role of imine bonds in capturing AuCl_4_^–^ was particularly evident in the recently reported PYTA-TDTA-COF (Figure 5h), where the imine nitrogens act as binding sites for the gold ions.^110^ Similar to TzDA-COF, the TTASDFP COF reported by the Trabolsi group features a triazine-based amine framework (Figure 4f),^79^ with ordered thioanisole groups within the pores. The COF functionalization and design were crucial for gold binding at ultralow concentrations. More importantly, the synthesis of the material is relatively easy, as it is conducted via microwave irradiation. This can be attributed to the reversibility of the imine bond and the generally mild reaction conditions needed for the formation.^111^ In addition, the significance of imine bonds in the overall COF stability is noted with SCOFs constructed by Liu et al. through the solvothermal Schiff base reaction of 1,3,5-triformylbenzene and 4,4-diaminophenyl sulfide.^112^ In this study, researchers showed the material‘s stability after treatment with NaOH, HCl, and other organic solvents, such as DMSO and THF. Imine-based COFs can also be structurally tailored with macrocycles for optimized adsorption. This is demonstrated with COF-V, which was formed by the reaction between 2,5-divinylterephthalaldehyde and 1,3,5-tris­(4-aminophenyl)­benzene.^78^ The COF was then enriched with cyclodextrin and dithiothreitol units to obtain COF-V-DTT and COF-V-S-β-CD (Figure 5l,m). These modifications endow them with stability and with hydroxyl and sulfhydryl groups that contribute greatly to the gold uptake.

#### 4.1.2 Keto-Enamine Linkage

The keto-enamine bond is formed through irreversible enol-keto tautomerization after Schiff base condensation, and it is known for enhancing the thermal and chemical stability of the material.^114^ This high stability makes β-ketoenamine-based COFs suitable for gold recovery under harsh conditions, such as highly acidic e-waste leaching solutions.^115^ For instance, MTpPa-1 composites demonstrated excellent AuCl_4_^–^ adsorption performance at pH = 2.0 while relatively maintaining crystallinity after 15 regeneration cycles using a 0.1 mol L^–1^ mixed solution of HNO_3_ and thiourea.^81^ This efficient adsorption is attributed to the carbonyl and −NH groups originating from the TpPa-1 COF component, synthesized via 2,4,6-triformylphloroglucinol (Tp) and primary amine condensation.^116^ Similarly, MoS_2_-TpTa and TpPa@Nb_2_C composites exhibited high stability in acidic conditions due to the β-keto-enamine TpTa COF formed using the Tp linker.^77,117^ More notably, MoS_2_-TpTa has shown strong potential in real e-waste leaching solutions, demonstrating its promise for applications on a wider scale. Another promising example is the Tp-BTD COF isomers,^71^ synthesized through Tp and benzothiadiazole-based amine (BTD) condensation. These isomers adopt different stacking models, AA, AB, and ABC, due to varying synthetic conditions. Generally, these COFs exhibit high stability, particularly Tp-BTD-AA (Figure 5k), which retained its crystallinity over a pH range of 1–9, making it highly suitable for gold recovery from e-waste. This is mainly because of its negative redox potential and superior surface area compared to the other isomers.

#### 4.1.3 Hydrazone Linkage

Hydrazone bonds (C=N—N) are constructed through the condensation of aldehydes with hydrazines or hydrazides and are known for providing stronger hydrolytic stability in COFs compared to imine linkages.^118^ Additionally, hydrazone linkages introduce synthetically modifiable heteroatomic sites that can pose as binding sites, which opens the door for versatile COF design.^119^ Additionally, due to the binding sites, hydrazone-linked COFs have been reported to capture heavy metal ions, including gold.^120^ A notable example is TTB-COF, developed by Zhou et al. via the reaction of 1,3,5-triformylbenzene and thio-based terephthalohydrazide (Figure 5e).^67^ These hydrazone linkages in the COF participate in the extended π-conjugation, which endows the material with fluorescence properties that are crucial in gold sensing applications. TY-Hz COF (Figure 5b) is another hydrazone-linked COF that the Qiu group prepared through the reaction of a pyrene-based aldehyde and a dihydrazide.^65^ Similar to TTB-COF, this COF exhibits a π-conjugated framework and fluorescence properties that can be exploited in gold sensing. Another hydrazone-linked COF is TTR-COF, which was designed with triazine and thioether-functionalized units.^113^ Interestingly, not only did it successfully recover gold ions from water, but the gold-enriched COF also demonstrated catalytic hydrogen production from seawater, highlighting its multifunctionality.

### 4.2 sp^2^-Carbon Linkages

sp^2^-carbon-conjugated COFs are noted for their high crystallinity, exceptional chemical stability, and extended π-conjugation, making them robust materials for gold recovery.^121,122^ One example is triphenylene-based COFs, namely COP-TPC6 and COP-TPC8, which incorporate flexible dibenzo-crown ethers.^103^ This design helps maintain structural integrity in acidic and neutral conditions (pH 3–7). Moreover, the C—O groups of the crown ethers act as binding sites for [AuCl_4_]^−^. Another example is the vinylene-linked JNM-100-AO, a hybrid structure bridging MOF and COF chemistry.^123^ This material is composed of Ag­(I) cyclic trinuclear units and triacetonitrile-based organic linkers. Due to its strong C=C bonds and the subsequent π-conjugation, JNM-100-AO demonstrates photoluminescent properties and exceptional stability in both highly acidic and basic media (pH 0–15), which are important for effective gold ion sensing and uptake applications. Moreover, the Ag­(I) units and the amidoxime functionalization offer binding sites for the gold ions. Overall, sp^2^-carbon-based COFs are endowed with the advantageous properties of enhanced chemical stability, rich π-electron systems, and luminescence activity due to the unique electronic properties.^122,124^ This underlines the premise of this synthetic strategy.

## 5 COF Functionalization and Composite Engineering for Gold Recovery

Based on the structural design and composition, COF materials reported for gold ion capture can be classified into functionalized COFs and COF-based composites. The latter incorporate other organic or inorganic components, enabling the composites to leverage the intrinsic advantages of COFs, such as porosity and tunability, while enhancing other properties, such as stability and durability.^125^ Both types of materials show overall improved adsorption performance, making them highly effective for recovering gold from waste leachates and other sources.

### 5.1 Functionalized COFs

Integration of functional groups into COFs is a powerful strategy for adjusting their physical and chemical properties, as well as increasing their selectivity and binding strength toward gold ions. Functional groups are typically introduced through two main pathways, presynthetic functionalization and postsynthetic modification (Figure 6a,b).^127,128^ Presynthetic functionalization is a bottom-up approach where the organic building units containing the desired atoms and moieties are integrated into COFs during synthesis. Examples include the ionic-COF-Cl, TTASDFP, and ECUT-COF-29 (Figure 7), which contain hydroxyl, amino, thioanisole, thioether, and other cationic groups known for their strong Au^3+^ binding through coordination and electrostatic interactions. On the other hand, postsynthetic modification involves incorporating the required functional groups into the surface or pore walls of the preformed COFs. This can be achieved via group transformation, linker exchange, and click reactions.^80,129^ This functionalization strategy offers the advantage of adding molecular units or groups that are challenging to introduce during initial COF synthesis. An example is the building block exchange of 4,4′-biphenyldicarboxaldehyde in TzBA COF with terephthaloyl chloride blocks to form the irreversible amide in JNU-1 COF.^80^

**Figure 6:**
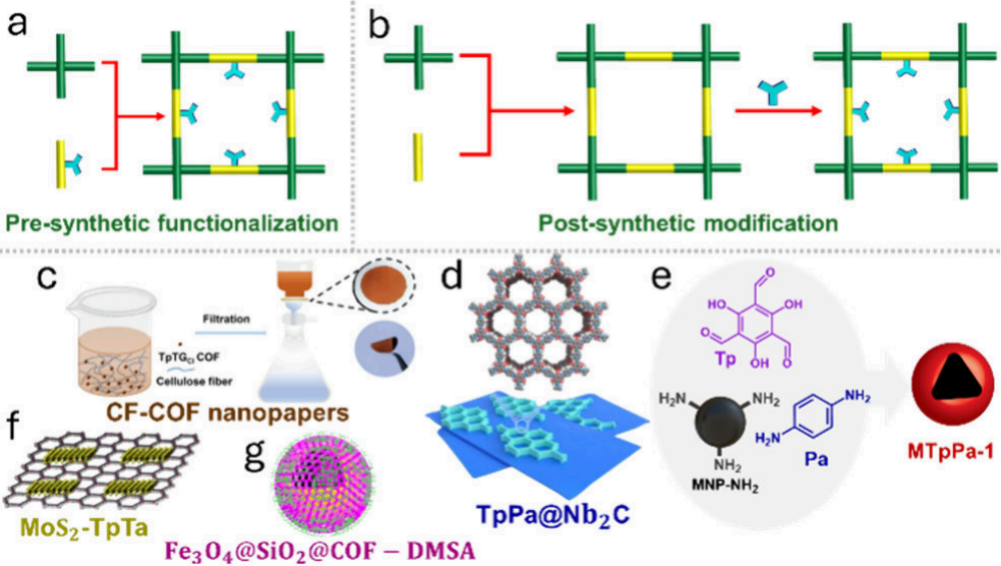
General formation routes of a, b) functionalized COFs and c–g) the structures of COF-based composites designed to remove gold ions from water. c) Adapted from ref 92. Available under a CC-BY 4.0 license. Copyright 2023 Xu et al. d) Adapted with permission from ref 117. Copyright 2024 Elsevier. e) Adapted with permission from ref 81. Copyright 2023 Elsevier. f) Adapted with permission from ref 77. Copyright 2024 Elsevier. g) Adapted with permission from ref 126. Copyright 2025 Elsevier.

**Figure 7:**
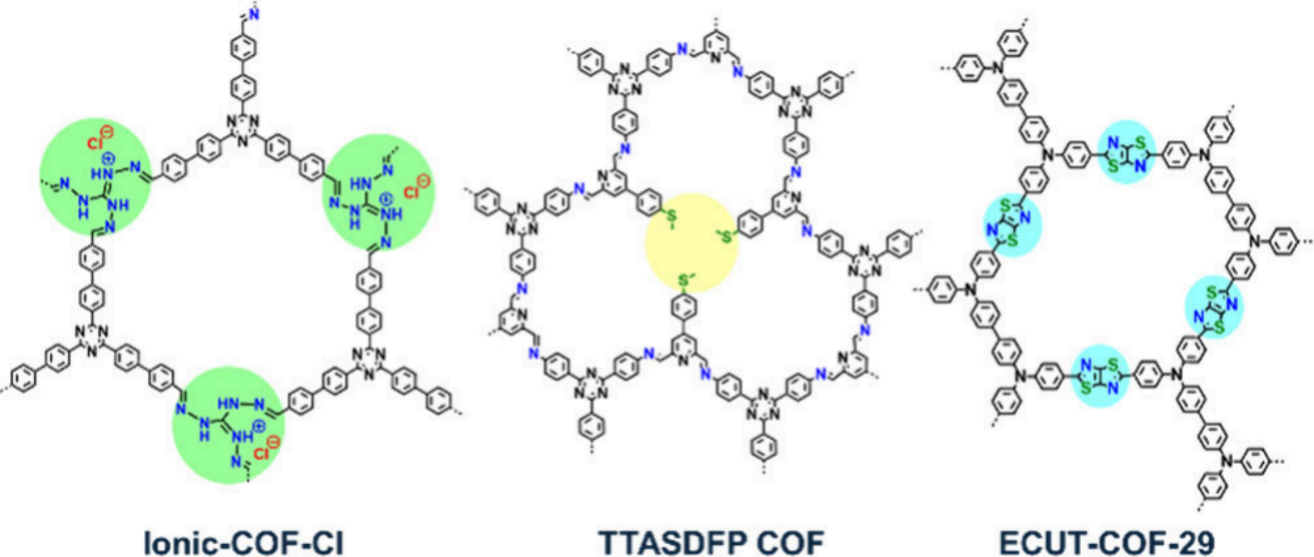
Structures of functionalized COFs with highlighted moieties that selectively bind Au^3+^.^22,66,79^

### 5.2 COF-Based Composites

These hybrid materials are constructed by combining COFs and other material components, such as metal oxides, silica, polymers, and carbon materials.^125,130^ This integration can make the functionality and structural stability of composites superior to that of COF components. This makes them a material of choice for gold recovery applications (Figure 6c). A notable example is CF-COF nanopapers, which consist of ionic guanidinium-based TpTG_Cl_ COF and cellulose fibers (CF).^92^ This composite was able to adsorb AuCl_4_^–^ from e-waste leaching solutions at a pH of 2.0. More importantly, the CF-COF membrane can be easily separated from solutions, making the recovery application more practical. Other COF-based composites with gold recovery capabilities include TpPa@Nb_2_C and MoS_2_-TpTa.^77,117^ TpPa@Nb_2_C was formed by the interfacial self-assembly of TpPa COF on niobium carbide (Nb_2_C) Mxene, while MoS_2_-TpTa was prepared by in situ growth of TpTa COF on molybdenum disulfide (MoS_2_). The MXene and MoS_2_ substrates introduce hydroxyl and sulfur functional groups, which contribute to gold uptake performance by improving the binding to the metal ions. Magnetic COF composites, such as Fe_3_O_4_@SiO_2_@COF-DMSA and MTpPa-1, are also promising gold adsorbents.^81,126^ Both materials consist of Fe_3_O_4_@SiO_2_–NH_2_ nanoparticles (MNP-NH_2_), which provide superparamagnetism and physicochemical stability. In addition, the oxygen groups in the materials facilitate gold ion adsorption from water. These materials can also be easily recovered from aqueous solutions through magnetic separation, making them highly practical, as demonstrated in the case of CF-COF nanopapers.

## 6 Gold Adsorption, Recovery, and Detection Using COFs

Efficient strategies for gold recovery from secondary sources, such as industrial wastewater and e-waste, are essential for gold sustainability and conservation. As mentioned, COFs and COF-based composites have shown significant gold adsorption potential. Their excellent gold capture performance is due to fast kinetics, high uptake capacity, selective gold adsorption from e-waste leachates, ultrasensitive detection, and gold capture from ultralow concentrations.

### 6.1 Rapid Gold Adsorption Kinetics

Some of the discussed COF-based materials demonstrated remarkably fast gold adsorption equilibrium times, ranging from seconds to a few hours, with the fastest being 10 and 30 s for JNU-1 and TTASDFP COFs, respectively (Figure 8).^79,80^ This resulted from the irreversible amide linkages in JNU-1 that provide strong C­(N)–H···Cl hydrogen bonds and Au–O coordinate bonds. In the case of TTASDFP, the fast equilibrium time was attributed to the thioanisole groups located within the pores, which serve as strong gold binding sites. Moreover, the ordered and porous nature of both COFs regulates and enhances the metal ion diffusion to the adsorption sites, ensuring rapid ion uptake.^131^ The kinetic studies also revealed that COFs significantly outperform other classes of adsorbents, such as MOFs, polymers, and biomass materials, which often require several hours or even days to reach adsorption equilibrium due to their limited surface area, porosity, or selectivity toward the gold ions.^132,133^

**Figure 8:**
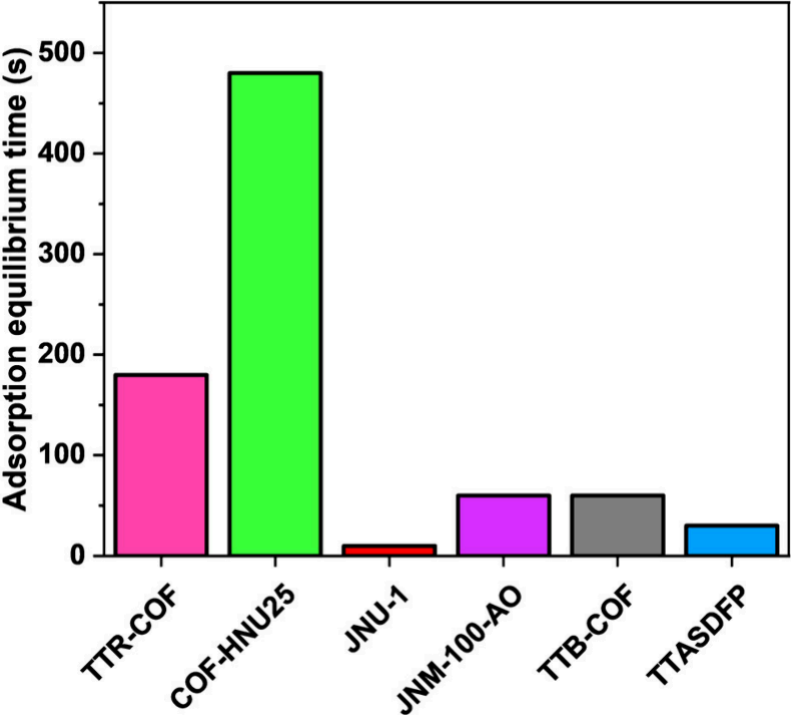
Most rapid Au^3+^ adsorption equilibrium times reported for COFs.^67,79,80,94,113,123^

### 6.2 High Gold Uptake Capacity

Aside from fast kinetics, COFs have also demonstrated high gold adsorption capacities, often exceeding 1000 mg g^–1^ (Table 1). Two of the highest reported capacities corresponded to TpTSc COF and Tp-BTD-AA at 4400 mg g^–1^ and 3094 mg g^–1^, respectively. These remarkable adsorption performances are mainly attributed to the well-distributed nitrogen, sulfur, and oxygen functional groups that interact strongly with gold ions. Additionally, the synergistic effect of in situ gold ion reduction on some COFs further contributes to their high adsorption capacity.^98^ These findings highlight the role of rational COF design and chemical functionalization in maximizing gold adsorption capacity.

**Table 1:** Comparative Analysis of Au^3+^ Adsorption Performance Recorded for COFs and COF-Based Composites, Consisting of the Adsorption Capacity, the Coefficients Obtained from the Adsorption Kinetic Fitting by the Pseudo-Second-Order Model, and the Regeneration Cycles

**Material**	**Maximum uptake**, ***q***_***m***_ **(mg g** ^**–1**^ **)**	**Pseudo-second-order coefficient**, ***k***_**2**_ **(g mg** ^**–1**^ **min** ^**–1**^ **)**	**Regeneration cycles**	**Reference**
JNU-1	1124	1.04	4	^80^
SCOFs	100	0.0014	4	^112^
COF-HNU25	1725	0.0061	20	^94^
TY-Hz COF	1008	0.0012	5	^65^
TTB-COF	560	-	4	^67^
TpTSc COF	4400	2.993 × 10^–4^	6	^82^
TpTG_Cl_ COF	1895	9.6885 × 10^–5^	3	^92^
MTpPa-1	1737	8.94 × 10^–3^	15	^81^
TzDa-COF	1866	-	4	^109^
N^+^-PYTA-PATA-COF	1834	4.73 × 10^–4^	10	^110^
PYTA-BDTA-COF	1752	4.83 × 10^–3^	-	^110^
Tp-BTD-AA	3095	0.0126	3	^71^
COF-V-S-β-CD	821	0.0032	3	^78^
MoS_2_-TpTa-1.5	2565	2.67 × 10^–5^	5	^77^
JNM-100	708	0.07778	5	^123^
JNM-100-AO	954	0.17220	5	^123^
TpPa@Nb_2_C	910	0.00025	3	^117^
TTASDFP	245	-	5	^79^
BMTA-TFPM-COF	570	-	5	^134^
ECUT-COF-29	3714	-	10	^22^
Fe_3_O_4_@SiO_2_@COF-DMSA	1116	33.78	6	^126^
Ionic-COF-Cl	1271	1.36 × 10^–3^	10	^66^

### 6.3 Selectivity and Reusability

In addition to high adsorption capacity, COF-based adsorbents demonstrate exceptional selectivity for gold ions, even in the presence of several competing metal ions such as Cu^2+^, Zn^2+^, Co^2+^, Pb^2+^, Fe^3+^, and Cd^2+^, with gold selectivity percentage often exceeding 97%. Moreover, COFs are reported to be highly durable and regenerable. This was supported by the reported regeneration cycles that ranged from 3 to 20 cycles (Table 1). Notably, some of the COFs were regenerated under mild conditions, such as exposure to thiourea,^94,110^ which highlights their general robustness and signifies their promising reusability. These results confirm that COFs are not only effective but also offer sustainable solutions to conserve natural gold resources and potentially valorize gold from waste on an industrial scale.

### 6.4 Gold Recovery from e-Waste and Environmental Samples

E-waste is the most prominent secondary source of gold, containing up to ten times more gold than natural ores.^30^ However, e-waste also contains various coexisting metals, such as Cu, Ag, Ni, Fe, Pb, and Al, which account for more than 60% of the waste’s content.^135,136^ This complex composition makes selective gold recovery from e-waste challenging. In hydrometallurgy processes, e-waste is typically digested in aqua regia, a highly corrosive leaching mixture of concentrated nitric and hydrochloric acids, to dissolve gold and other metals into solution.^137^ The challenge lies in selectively extracting gold from this highly acidic, metal-rich leachate. Fortunately, the high stability of COFs in acidic environments and high selectivity for gold promote the successful recovery of gold from e-waste.

COF-based adsorbents have been tested on real e-waste samples, such as printed circuit boards (PCBs) and central processing units (CPUs), as well as simulated e-waste leachates that mimic real-world metal compositions.^109^ Notably, these COF materials achieved gold removal efficiencies of approximately ≥ 90% (Table 2). Moreover, the removal is selective as minor percentages, often <10%, of the other coexisting ions, such as Cu^2+^ and Ni^2+^, are adsorbed. These results emphasize the great potential of COFs in selective gold recovery from e-waste. In addition, COFs demonstrated the ability to recover gold from flow systems, which provides operational efficiency during industrial gold recovery. For instance, TzDa-COF displayed an efficient gold adsorption performance from a simulated e-wastewater system at a flow rate of 3 mL min^–1^.^109^

**Table 2:** Comparison of Gold Removal from Real and Simulated Electronic Waste Leachates via COF-Based Materials

**Material**	**Au** ^**3+**^ **conc. (ppm)**	**Leaching agent**	**Removal %**	**Reference**
CF-COF	8.92	Aqua regia	90.3	^92^
Tp-BTD-AA	3.67	N-bromosuccinimide (NBS) and pyridine solution	97.8	^71^
TpTsc	81.8	-	99.69	^82^
TzDa-COF	80	-	99	^109^
COF-V-S-β-CD	18.3	Aqua regia	99.2	^78^
MoS2-TpTa-0.5	15.01	Aqua regia	99.93	^77^
MoS2-TpTa-1.5	15.01	Aqua regia	100	^77^
Ionic-COF-Cl	18.92	Aqua regia	99.5	^66^
TY-Hz COF	81.8	Leaching solution	89.1	^65^

In addition to real and simulated e-waste, researchers explored gold recovery from real and simulated wastewater and seawater. This was best showcased with JNM-100-AO, in which the material was subjected to wastewater obtained from the kitchen water.^123^ In this test, 99% of the present 20 ppb Au^3+^ was extracted within 3 min. JNM-100-AO was also tested with seawater sourced from the South China Sea and spiked with gold to obtain 20 ppb Au^3+^. The COF material successfully captured 95% of the gold ions within 10 min, which underlines the adsorbent’s high selectivity toward gold ions in a complex aqueous environment containing organic compounds, microorganisms, dissolved salts, and other substances.^138^ Another example is TTR-COF, which reported the selective adsorption of gold ions against other alkaline and alkaline metals found in seawater under 3.5% and 5.0% salt.^113^ Despite the great premise of these reports, research in this area remains relatively limited.

### 6.5 Detection and Removal of Trace Levels

In addition to the e-waste, a substantial amount of gold is found in various water bodies, such as seawater, rivers, industrial sewers, and wastewater.^139,140^ However, the large water volume dilutes the metal concentration to trace levels as low as 10 ng L^–1^,^28^ making it highly challenging to detect and extract these gold traces.

Owing to their unique physicochemical properties, COFs were explored for both detecting and capturing ultralow trace amounts of gold. An example is TY-Hz COF, which showcased the dual function of gold ion detection and adsorption.^65^ Due to its hydrazone linkages and unique fluorescent properties, the π-conjugated COF effectively detects gold, which was measured through the quenched fluorescence peak at 530 nm induced by electron transfer. The COF achieves 87% fluorescence quenching within 10 s of exposure to 30 μM Au^3^ (Figure 8a). Moreover, the quenching activity is highly selective to gold ions in the presence of competing metal ions, such as Zn^2+^, Ni^2+^, and Pb^2+^. TTB-COF and JNM-100 also demonstrate strong and selective Au^3+^ sensing capabilities (Figure 8b,c).^67,123^ The fluorescence intensity of TTB-COF at 540 nm decreased by 84% upon exposure to 10 μM Au^3+^ (Figure 8b). On the other hand, JNM-100 achieved 99% fluorescence quenching under identical conditions. However, JNM-100 recorded a smaller limit of detection (LOD) of Au^3+^ compared to both TTB-COF and TY-Hz COF (Table 3). The strong fluorescence quenching for J NM-100 can be attributed to the metal–metal interactions between Au^3+^ and Ag­(I) units.

**Table 3:** Gold Detection Limit via COF-Based Materials, Calculated through the Fluorescence Quenching

**Material**	**Au** ^**3+**^ **LOD**	**Reference**
TY-Hz COF	17 nM	^65^
TTB-COF	1.39 μM	^67^
JNM-100	60 ppb	^123^
JNM-100-AO	66 ppb	^123^

Beyond sensing, COFs have shown promising capabilities in capturing gold ions from concentrations as low as ppb, which is evident with the TTASDFP-COF.^79^ The integrated and closely arranged sulfur-based groups in the COF’s pores served as chelating sites that can extract gold ions from concentrations ranging from 16 to 2 ppb (Figure 9d,e). This adsorption performance is maintained even in saline solutions containing >10,000 ppm of Na^+^, simulating wastewater environments (Figure 9f). TTASDFP-COF also captured gold in the presence of an excess of competing soft metals, such as copper (Figure 9g). These COFs illustrate the impact of structural design and functionalization in endowing the COFs with high sensitivity and selectivity toward trace concentrations of gold ions.

**Figure 9:**
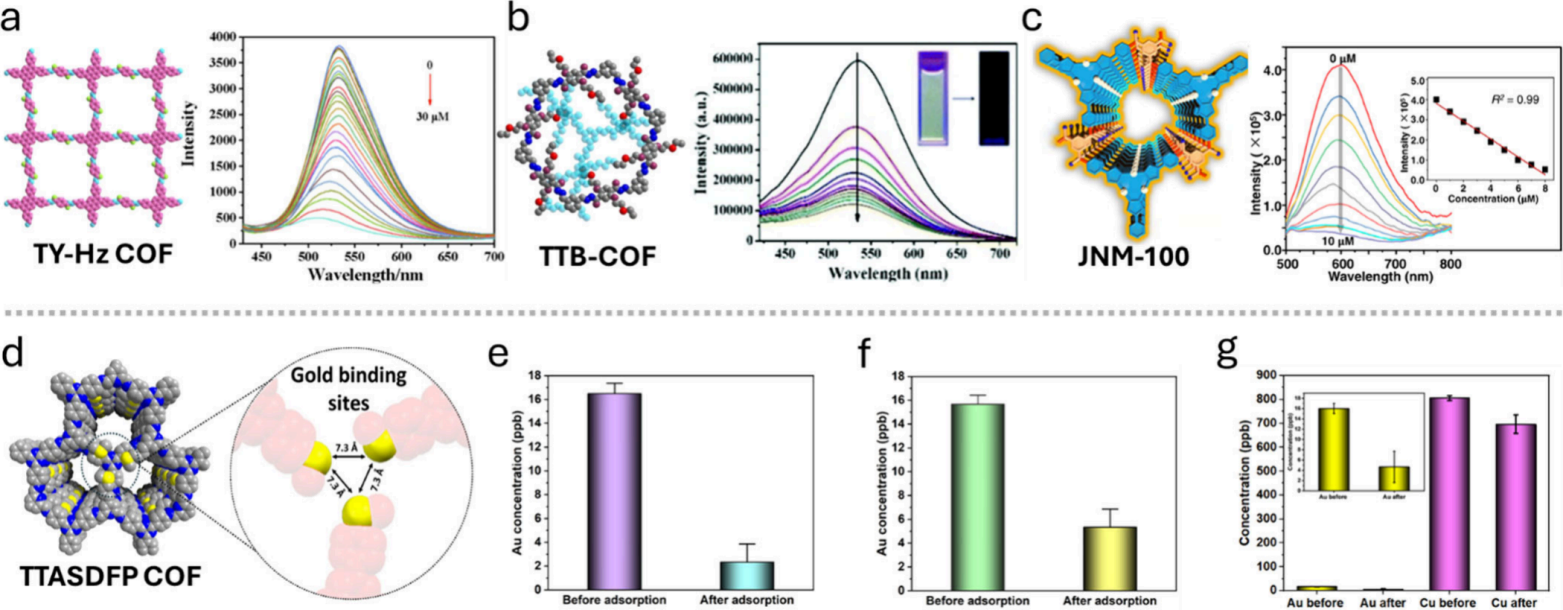
Detection and capture of ultralow trace levels of gold via different COFs. a–c) Fluorescence spectra of TY-Hz COF, TTB-COF, and JNM-100 in the presence of increasing concentrations of Au^3+^ (0–30 μM, 1–10 μM, and 0–10 μM, respectively). a) Adopted with permission from ref 65. Copyright 2023 Elsevier. b) Adopted with permission from ref 67. Copyright 2018 Royal Society of Chemistry. c) Adapted from ref 123. Available under a CC-BY 4.0 license. Copyright 2022 Luo et al. d) The structure of TTASDFP COF’s pores shows the sulfur binding sites. e) The concentration of Au^3+^ before and after exposure to TTASDFP suspension in a NaCl-free system. f) Solution containing 10800 ppm of Na^+^. g) The concentration of Au^3+^ and Cu^2+^ in a solution containing 10,800 ppm of Na^+^ before and after adsorption via TTASDFP COF. d–g) Adapted from ref 79. Available under a CC-BY 4.0 license. Copyright 2025 Abubakar et al.

## 7 Evaluating the Gold Adsorption Performances of Other Materials

Aside from COFs, several other materials have been reported for the adsorption and recovery of gold ions from water, primarily MOFs, polymers, and other inorganic materials. Notably, some of these materials displayed great gold uptake capacities that can exceed 3000 mg g^–1^, as reported for FeCO-MOF-74 (Figure 10).^141^ Some of the MOFs and composite-based materials also displayed considerable gold adsorption capacities, with the example NH_2_-UiO-66-BA (2040 mg g^–1^) and phenylenediamine (PmPD) nanoparticles (2040 mg g^–1^).^142,143^ Similar to COFs, the adsorption of the Au^3+^ via MOFs and other adsorbents is mainly facilitated via electrostatic interactions and coordination bonding.^53,142–155^ However, although these uptake capacities can rival those showcased for COFs, the gold recovery efficiency of many MOFs and organic/inorganic materials remains significantly limited by the slow adsorption kinetics, with some recent examples (Figure 10) reporting adsorption equilibrium time in the range of 1 min to 10 h.

**Figure 10:**
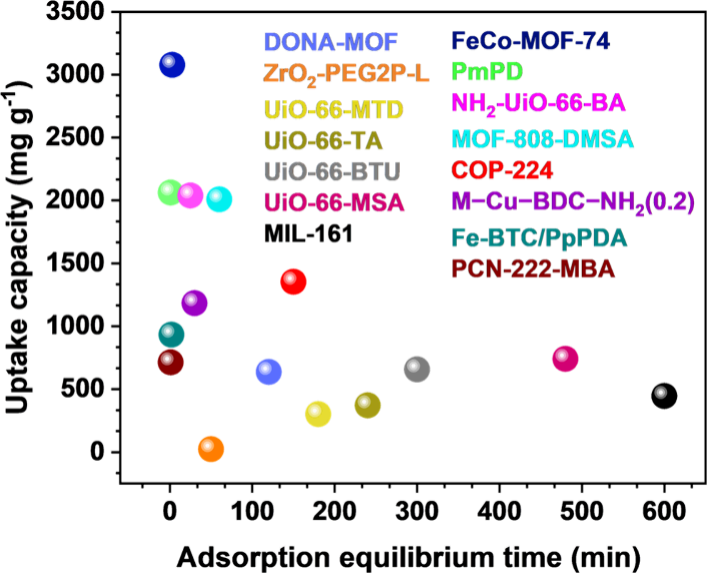
Comparison of the gold adsorption capacity and kinetic equilibrium time for some of the MOFs, polymers, and inorganic-based adsorbents reported during 2018–2025.^53,142,144,146–154,156^

## 8 COFs for Gold Recovery: Challenges and Future Outlooks

The extraction and recovery of gold from aqueous solutions represent a sustainable strategy for conserving this precious resource. Extensive research has been dedicated to enhancing adsorption efficiency through the design of COF-based adsorbent materials, paving the way for a promising future in gold extraction. Despite significant advancements, several challenges persist in using COFs for wide-scale gold recovery. A primary hurdle is the limited scalability of COF-based gold adsorbents, as most are synthesized using laboratory-scale techniques that demand specific conditions and prolonged reaction times, complicating large-scale production.^129^ Addressing this issue involves understanding and streamlining the materials‘ synthetic procedures, as demonstrated by the MTpPa-1 COF, which was synthesized through manual grinding within 30 min; a rapid, cost-effective, and eco-friendly approach.

Moreover, there are other recent efforts made to achieve scalable COF synthesis. This includes flow-based synthesis. This emerging synthesis strategy is conducted through the continuous flow of the reagents and reactants, as opposed to the conventional batch synthesis. This provides the advantages of increased reaction efficiency due to high throughput, and controlling reaction conditions, including flow rate and residence time.^157,158^ While flow-based synthesis has been applied to COFs,^159,160^ its potential for large scale gold recovery remains unexplored. Other viable COF synthesis strategies are aqueous-phase synthesis and the solvent-free method, which are based on avoiding the use of organic solvents to minimize waste and secondary pollution, in addition to simplifying the synthesis and post-treatment processes.^161^ The solvent-free strategy is particularly promising considering that prototypical synthetic methods, such as solvothermal, depend on the reactants’ solubility, the solvent-reactants ratio, the catalyst concentration, and other solvent-related reaction conditions.^162^ Mechanical methods are also considered common approaches for COF synthesis, and they are increasingly adopted in industry in general.^163^ This is mainly due to easy operation and energy efficiency, making scale-up synthesis feasible. Through continued research on sustainable and eco-friendly synthesis methods, scalable COFs can become more accessible, unlocking the full potential of these materials as practical gold adsorbents.

Another challenge facing COF-based gold adsorbents is the limited number of studies involving real water samples, as most gold adsorption studies were conducted under controlled laboratory conditions. This is due to the complex composition of real gold leachates or waste streams, in which substances such as earth metals and organic impurities are present.^164^ Moreover, factors such as pH variations and temperature differences also complicate adsorption processes. Although COFs have shown high selectivity toward gold and have been tested on simulated e-waste samples,^109^ further efforts are needed to optimize COF structures and enhance their efficiency in treating industrial and environmental samples. Future research should aim to improve COF stability and selectivity in complex matrices, bridging the gap between laboratory studies and large-scale applications.

The long-term durability of COFs is another critical factor in their viability as sustainable gold adsorbents. While many of the COFs discussed have demonstrated great stability and reusability, maintaining their structural integrity over several adsorption–desorption cycles, the advancement of efficient and environmentally friendly COF regeneration methods remains an active area of research. One example is the use of thiourea leaching,^79,80^ which is considered less toxic than conventional regeneration solutions, such as cyanide.^165^ Despite the advances in enhancing the overall COF stability through structural design, functionalization, and strategic choice of covalent linkages, some COFs face the challenges of hydrolytic and chemical stability, particularly in harsh acidic and basic environments. This is due to the reversibility principle of the COF bond formation that is crucial for the network crystallinity.^166^ Thus, to improve the COF stability, the main methods include limiting the bond reversibility via surface modification, tailoring the interlayer COF stacking forces, and adjusting the steric properties of the COFs.^167^ Another effective approach to address the limited durability of COF-based gold adsorbents is to develop more self-standing COF materials or continuous COF-based membranes, since they offer the unique advantages of mechanical integrity and practicality.^168^ which are essential for industrial-level gold recovery applications.

As research advances, new opportunities are emerging for COF-based gold recovery. Future studies should explore selective capture of various gold oxidation states, extending COFs’ utility beyond Au^3+^. Additionally, designing multifunctional COFs that integrate gold sensing, adsorption, and reduction capabilities for real-time monitoring should be a focus. Furthermore, exploring COF-based hybrid composites by combining COFs with nanomaterials, polymers, or biomolecules could enhance adsorption efficiency and stability in diverse environments. Progress in these areas could lead to widespread implementation of COFs in industrial gold recovery, environmental remediation, and sustainable resource management.

Moreover, to accelerate the rational design of COFs for gold detection and recovery, the integration of artificial intelligence (AI) and machine learning (ML) offers a powerful and transformative strategy. These computational tools can analyze vast data sets to uncover structure–property relationships, enabling the prediction of COF performance metrics such as adsorption capacity, selectivity, and stability under diverse environmental conditions. ML algorithms can guide the selection of functional monomers and optimal topologies by identifying patterns within existing COF data sets, significantly reducing experimental trial-and-error. Furthermore, AI-driven models can simulate interactions between gold species and COF frameworks in complex aqueous matrices, allowing researchers to anticipate potential interferences and optimize COF chemistry accordingly. By incorporating AI/ML approaches into the materials discovery pipeline, the design of next-generation COFs can become more targeted, efficient, and performance-driven, thereby accelerating their translation into real-world gold recovery applications.

Overall, the recent advances in overcoming the previously discussed challenges significantly impact the environmental and lifecycle profiles of COF-based gold adsorbents. These materials offer strong potential for sustainable applications, as they are topically synthesized from biocompatible organic linkers composed of light elements, often under milder, more eco-friendly conditions.^161^ This reinforces their suitability for water remediation, expanding their environmental value.^169^ On the other hand, the lifecycle implications of COFs, such as usability, recyclability, manufacturing costs, and waste generation during synthesis, remain important considerations.^170^ These challenges can be mitigated through more sustainable synthetic strategies such as mechanochemical synthesis, aqueous-phase condensation, and postsynthetic modifications—can further reduce environmental impacts and lower costs. Furthermore, integrating life cycle analysis (LCA) into material development helps systematically identify environmental hotspots and design remediation strategies from the outset. The continued refinement of COF synthesis and lifecycle management will advance their environmental performance and economic feasibility, supporting their broader adoption for sustainable gold recovery and other remediation applications.

In order to ensure the practicality and effectiveness of COF materials as gold adsorbents, standardized testing protocols need to be drafted and established. Generally, the testing protocols of the characterized adsorbents involve batch adsorption tests, which are implemented in research laboratories, pilot-scale investigations to evaluate the suitability at an industrial level, and testing using commercial columns.^171,172^ These protocols serve as guidelines to evaluate and develop adsorbents targeted for various water contaminants. To date, there has been some progress in proposing porous materials for adsorption and water treatment.^171^ However, much work remains to establish protocols for COFs tailored for the recovery of precious metals, such as gold, from water. Nevertheless, current advances in this area indicate a promising outlook for COF-based gold adsorbents.

## 9 Summary and Conclusions

This review presents an in-depth examination of COF-based materials utilized in the sensing, adsorption, and recovery of gold ions from aqueous solutions, providing a sustainable and cost-effective alternative to traditional gold recovery techniques. Initially, we delved into the fundamental mechanisms governing gold adsorption, such as coordination bonding, electrostatic interactions, and hydrogen bonding. A thorough understanding of these interactions is crucial for guiding the strategic design of COFs to optimize their performance in selective and efficient gold recovery. The review then offers an overview of various COF synthetic methodologies and the strategies employed in their structural design, including polymerization reactions, postsynthetic modifications, and functionalization with gold-binding atoms like nitrogen and sulfur.These approaches enable precise control over COF architecture and surface chemistry. Subsequently, we highlight the gold adsorption performances from both e-waste leachates and solutions containing ultralow traces of gold. The reported uptake capacities of COFs surpass those of many other organic and polymeric materials. Furthermore, the high selectivity of COFs for gold, even amidst competing metal ions, underscores their significant potential for industrial-scale gold recovery.

Despite these promising advancements, challenges remain. Scalability is a major hurdle, as COF synthesis typically occurs on a laboratory scale and can be resource-intensive. Additionally, most studies have been conducted under controlled conditions, necessitating further optimization for real-world applications. Future research should prioritize gold recovery from real water samples by enhancing the durability, selectivity, and regeneration capabilities of COFs. This can be achieved through the development of cost-effective synthesis and regeneration techniques, along with the design of novel multifunctional COFs that integrate sensing, adsorption, and gold ion reduction capabilities.

Ultimately, overcoming current limitations and leveraging emerging technologies could pave the way for next-generation COF-based platforms, enabling sustainable, scalable, and highly efficient gold recovery solutions.
